# Longitudinal change of energy expenditure, body composition and dietary habits in Progressive Supranuclear Palsy patients

**DOI:** 10.1007/s10072-024-07533-5

**Published:** 2024-04-19

**Authors:** Maria Francesca Tepedino, Anna Rosa Avallone, Filomena Abate, Marina Serio, Miriam Caterino, Roberto Erro, Maria Teresa Pellecchia, Paolo Barone, Marina Picillo

**Affiliations:** 1 Center for Neurodegenerative Diseases (CEMAND), Department of Medicine, Surgery and Dentistry “Scuola Medica Salernitana”, University of Salerno, 84131 Salerno, Italy; 2 Department of Medicine, Surgery and Dentistry “Scuola Medica Salernitana”, Via Allende, 84081 Baronissi, Salerno, Italy

**Keywords:** Progressive Supranuclear Palsy, Rest energy expenditure, Body composition, Dietary habits, Proteins, Dysphagia

## Abstract

**Introduction:**

Alterations in metabolic status, body composition, and food intake are present in all neurodegenerative diseases. Aim of this study was to detect the progression of these changes in Progressive Supranuclear Palsy (PSP).

**Methods:**

We conducted a longitudinal study of 15 patients with PSP. The assessments were performed at baseline (T0) and after 7(IQR = 5) months of follow-up (T1). We collected anthropometric measures including body weight, height, body mass index and waist circumference, metabolic parameters through indirect calorimeters, body composition using bioimpedance analysis, and dietary habits with a validated questionnaire. PSP-rating scale (PSP-rs) was used to evaluate disease severity and dysphagia.

**Results:**

The majority of patients (66.66%) presented PSP-Richardson Syndrome and 33.33% the other variant syndromes of the disease. At T1 there was a decrease in intake of total daily calories (*p* < 0.001), proteins (*p* < 0.001), fibers (*p* = 0.001), calcium (*p* = 0.008), iron (*p* < 0.001), zinc (0.034), vitamin E (*p* = 0.006) and folates (*p* = 0.038) compared to T0. No other changes were found. As for T1 data, no significant differences were shown according to disease phenotypes or the presence of clinically significant dysphagia for solids.

**Conclusions:**

Within a mid-term follow up, PSP patients presented reduced caloric and proteins intake regardless the presence of dysphagia. The PSP-rs is likely not adequate to assess dysphagia, which should be investigated by specific clinical scales or instrumental examinations. With the goal of maintaining adequate nutritional status, the administration of protein and vitamin supplements should be considered even in the absence of dysphagia evidenced by the rating scales.

**Supplementary Information:**

The online version contains supplementary material available at 10.1007/s10072-024-07533-5.

## Introduction

Changes in the metabolic state, body composition and dietary intake are present in all neurodegenerative diseases [1–3]. Resting energy expenditure (REE) represents the number of calories required by the body to maintain vital physiological functions and the state of wakefulness. It represents approximately 50–70% of the total energy consumed and it is influenced by individual factors, such as gender and age, as well as comorbidities [1–3]. Indeed, an hypermetabolic state, defined as increased REE, is considered linked to the process of neurodegeneration and it is likely responsible of weight loss in Amyotrophic lateral sclerosis (ALS), Frontotemporal Dementia spectrum, Alzheimer’s disease and in the advanced stages of Parkinson’s disease [1, 3]. Furthermore, changes in dietary habits associated with dysphagia are also responsible for weight loss during advanced stages of neurodegenerative diseases [4].

Progressive supranuclear palsy (PSP) is a rapidly progressive 4-R tauopathy characterized by significant weight loss since the earliest stages [5]. Recently, we conducted a cross-sectional study demonstrating that, irrespective of dietary habits, greater disease severity is associated with a reduction in REE and changes in body composition with lower fat-free mass (FFM). [5]. To date, there is a lack of longitudinal studies evaluating progression of metabolic changes in PSP. The aims of the present study were to explore longitudinal changes in metabolic state, body composition and dietary intake in PSP over mid-term follow up.

## Material and Methods

### Study population

PSP patients according to current criteria were enrolled at the Center for Neurodegenerative diseases (CEMAND) of the University of Salerno, Italy, between June 2018 and April 2023 and evaluated twice, at baseline and after a median of 7 months (Interquartile range, IQR = 5) [6, 7]. The project was approved by local Ethics Committee and all patients gave written informed consent.

All assessments were performed in the morning, after 12 h of fasting, after the regular dopaminergic treatment, in a quiet environment and with the patient in a sitting position.

Inclusion criteria were verified also at follow up and included the capability to stand up and walk at least 10 steps with or without unilateral support, the physical and cognitive ability to perform the required assessments, and the availability of a caregiver to support the compilation of the dietary habit questionnaire. Patients had stable dopaminergic therapy during the study.

Exclusion criteria were the presence of conditions with a known disease influencing metabolism and weight (eg, diabetes) and implant of pacemakers or other electrical device contraindicating the assessment of body composition [5].

## Assessments

Anthropometric measures including body weight, height, body mass index (BMI) and waist circumference were recorded. Metabolic parameters including REE and total daily energy expenditure (TDEE) were computed as detailed elsewhere and in supplemental material [5]. Finally, we estimated physical activity (PAL) intensity levels (sedentary or poor active or active or very active) from the ratio of TDEE to REE (supplemental material). FFM, fat mass (FM) and the total body water were estimated with bioimpedance analysis. Dietary habits were estimated with validated online freeware software developed by the Grana Padano Observatory, which provided information on daily intake of calories, macronutrients, micronutrients, water and alcohol (www.educazionenutrizionale.granapadano.it).

Information on age, disease duration and levodopa equivalent daily dose (LEDD) was collected for all patients. Disease severity was assessed with the PSP-rating scale (PSP-rs), the Movement Disorder Society version of the United Parkinson's disease III (MDS-UPDRS-III), and the Schwab and England (S&E); dysphagia for solids and liquids and the ability to use cutlery were collected with the corresponding items 3, 13 and 4 of the PSP-rs; cognitive skills were evaluated with the Montreal Cognitive Assessment (MOCA) [5].

## Statistical analysis

Shapiro–wilk test was run to determine suitability of variables for parametric or non-parametric analysis. Differences between paired group at baseline and follow-up were performed using paired t-test, signed-rank Wilcoxon or Mc Nemar test or χ-square, as appropriate.

Pre-specified sub-group analyses were performed for PSP phenotypes [Richardson’s syndrome (PSP-RS) versus the other variant syndromes of PSP (vPSP)] and clinically significant dysphagia for solid (PSP-rs item 3: < 2 versus ≥ 2).

Statistical analyses were performed using the Statistical Package for Social Science (SPSS version 26; SPSS, Inc., Chicago, IL). All statistical tests were two-tailed, and a *p*-value < 0.05 was assumed to be statistically significant.

## Results

A total of 15 PSP patients (10 men and 5 women) were included in the study and were evaluated at baseline and follow-up after 7 (5) months [median (IQR)]. Ten PSP patients (66.66%) presented PSP-RS and 5 vPSP (33.33%), of whom 3 with predominant parkinsonism and 2 with predominant frontal presentation. Five patients (33.33%) presented clinically significant dysphagia for solids.

Table 1 shows demographic, clinical and anthropometric features of the cohort study at baseline (T0) and follow-up (T1). As expected, at T1 patients presented greater total PSP-rs (*p* = 0.001) and MDS-UPDRS-III (*p* = 0.022) and lower S&E (*p* = 0.001) and MOCA (*p* = 0.004). Also, a greater proportion of patients presented clinically significant issues in using cutlery (PSP-rs item 4, *p* = 0.046) at follow up. No significant differences were detected for other variables.

**Table 1:** Demographic, clinical and anthropometric features of the PSP cohort

	T0	T1	*p*
Age, years	68.87(5.13)	69.80(4.57)	**0.010**
LEDD, mg/day	359.67(260.305)	336.67(246.74)	0.603
PSP-rs	35.80(10.65)	44.80(10.79)	**0.001**
PSP-rs item 3,^*^ n (%)	2(13.33)	5(33.33)	0.083
PSP-rs item 4,^*^ n (%)	6(40)	10(66.66)	**0.046**
PSP-rs item 13,^*^ n (%)	9(60)	12(80)	0.083
MDS-UPDRS-III	30.27(10.0)	41.87(16.02)	**0.022**
S&E	68.67(15.976)	54.00(16.818)	**0.001**
MOCA	20.21(5.31)	17.21(5.37)	**0.004**
Weight, Kg	80.540(12.0)	79.633(10.42)	0.640
Height, cm	166.00(10.80)	166.53(11.05)	0.543
BMI, kg/m2	29.31067(4.38)	28.9127(4.56)	0.487
BMI categories			
Normal weight, n (%)	2(13.33)	3(20)	0.655
Overweight, n (%)	8(53.33)	8(53.33)	1
Obese, n (%)	5(33.33)	4(26.66)	0.317
Waist circumference, cm median (IQR)	100(8)	103(11)	0.478

All data are expressed in mean (standard deviation), unless otherwise specified

Significant differences are highlighted in bold

Abbreviations: BMI: body mass index; LEDD: levodopa equivalent daily dose; MDS-UPDRS-III: the Movement Disorder Society version of the United Parkinson’s disease part III; PSP-rs: Progressive Supranuclear Palsy-rating scale; S&E: Schwab and England; ^*^number (%) of patients with score ≥ 2 at the corresponding item)

Table 2 reports comparisons between T1 and T0 for energy expenditure, body composition, and dietary intake. Only dietary intake presented a few significant changes at follow up. In detail, there was a decrease in intake of total daily calories (*p* < 0.001), proteins (*p* < 0.001), fibers (*p* = 0.001), calcium (*p* = 0.008), iron (*p* < 0.001), zinc (0.034), vitamin E (*p* = 0.006) and folates (*p* = 0.038).

**Table 2:** Study Assessments of the PSP cohort

	T0	T1	*p*
*Energy expenditure*			
Measured rest energy expenditure (mREE), Kcal/day	1686.20(542.84)	1683.0(399.90)	0.985
Total daily energy expenditure (TDEE), Kcal/day	2426.67 (651.09)	2475.70(582.89)	0.691
Physical activity level (PAL)	1.49(0.16)	1.48(0.10)	0.865
PAL intensity level, n (%)			
< 1.40 sedentary	4(26.66)	3(20)	0.705
1.40–1.69 low active	8(53.33)	11(73.33)	0.257
1.70–1.99 active	3(20)	1(6.66)	0.317
2.00–2.40 very active	0(0)	0(0)	1
*Body composition*			
Free fat mass, kg	61.29(9.8)	59.17(10.57)	0.299
Free fat mass index, kg/m	36.79(4.75)	35.44(5.25)	0.299
Fat mass, kg	18.77(12.39)	19.75(10.15)	0.639
Fat mass index, kg/m	11.34(7.77)	12.0(6.54)	0.599
Skeletal muscle mass, Kg	31.35 (8.28)	29.54 (8.29)	0.419
Skeletal muscle mass index, Kg/m2	11.33 (2.98)	10.56 (2.51)	0.411
Pathologic skeletal muscle mass index, n (%)	0 (0)	2 (13.33)	0.480
Body cellular mass, kg, median (IQR)	32.65(16.6)	27(9.9)	0.730
Body cellular mass index, kg/m, median (IQR)	19.25(7.3)	16.85(5.4)	0.826
Total body water, L	46.31(7.82)	44.79(8.46)	0.441
Total body water index, L/m	27.807(4.04)	26.793(4.28)	0.419
Extracellular water, L	20.70(3.65)	21.143(3.29)	0.372
*Dietary intake*			
Calorie intake, Kcal/day	1854.87(582.78)	1695.27(588.36)	** < 0.001**
Protein intake, g/day	74.16(25.11)	68.75(21.29)	** < 0.001**
Carbohydrates intake, % - Sugars, %	49.73(3.08) 21.53(6.56)	47.07(6.95) 22.73(7.49)	0.511 0.393
Lipid intake, % - SFA, %, median (IQR) - PUFA, %, median (IQR)	32.00(4.03) 11(3) 4 (2)	34.47(6.47) 11(3) 5(2)	0.330 0.217 0.465
Water intake, mL/day, median (IQR)	1400 (500)	1400 (600)	0.755
Fibers intake, g/day	30.42(13.17)	25.06(9.94)	**0.001**
Calcium intake, mg/day	997.80(349.04)	994.33(372.98)	**0.008**
Iron intake, intake, mg/day	12.87(5.535)	10.41(3.40)	** < 0.001**
Zinc intake, mg/day	10.38(4.56)	9.83(2.89)	**0.034**
Vitamin A intake, μg/day, median (IQR)	1088(999)	1037 (408)	0.307
Vitamin D intake, μg/day, median (IQR)	1.9(2.4)	2.3(2)	0.977
Vitamin E intake, mg/day	12.72(5.13)	11.15(3.78)	**0.006**
Vitamin B12 intake, mg/day	5.04(2.65)	4.74(1.80)	0.108
Vitamin C intake, mg/day	229.27(121.68)	208.67(105.09)	0.170
Folate intake, μg/day	414.47(196.93)	354.60(121.14)	**0.038**
Alcohol intake, g/day, median (IQR)	2.6(12.4)	1.7(10.3)	0.255

All data are expressed in mean (standard deviation), unless otherwise specified

Significant differences are highlighted in bold

Abbreviations: PUFA Poly-unsaturated fatty acids; SFA Saturated fatty acids

As for T1 data, no significant differences were shown according to disease phenotypes (table S1).

Patients with clinically significant dysphagia for solids presented a trend towards a lower intake of alcohol compared to patients without clinically relevant dysphagia for solids (*p* = 0.053) (table S2).

## Discussion

This is the first study assessing longitudinal changes in metabolic status, body composition and dietary habits in PSP. After a median follow up of 7 months, our patients presented stable REE, TDEE and PAL as well as body composition. Notwithstanding, we observed a significant reduction of daily calories, protein, fibers, calcium, iron, zinc, vitamin E and folate. Such pattern of change in both macro- and micronutrients suggest that as disease progresses PSP patients consume less frequently meat, vegetables, fresh and dried fruits, and legumes. However, such changes were not sufficient to determine variations in weight and body composition at mid-term follow up.

Recently, Cova et al. evaluated nutritional status in 40 patients with Alzheimer’s disease and failed to find significant differences compared with healthy controls as well as significant longitudinal changes over a follow up of 8.7 ± 3.6 months [8]. The short duration of follow up may in part account for the lack of significant changes in both studies and we can not exclude a longer follow up would allow revealing appreciable changes in PSP. However, as a matter of fact, PSP is a rapidly progressive disease and most patients may not be able to perform the study assessments after a longer follow up (eg, evaluation of REE with indirect calorimeter).

Reduction in intake of meat and vegetables may depend on a worse capacity of the oral phase during swallowing even in presence of a stability of the pharyngeal phase, which is indeed the focus of PSP-rs item 13. As a matter of fact liquid intake, which mostly rely on laryngeal phase, is stable at follow up. On the other hand, ingestion of solid food is more complex and guaranteed by a four-step process within the oral phase including mouth closure and retention of food, chewing, formation of the bolus by the muscles of the oral cavity with saliva, and placement of the bolus on the faucal pillars. All the steps of the oral phase are active in contrast with the pharyngeal phase which involves automatic movements. Dysfunction of the oral phase results in changes in eating habits and simplification of the diet, with a reduction in meat and vegetables (and thus protein, fibers and vitamins) and a stable proportion of carbohydrates and fats [9]. We speculate progressive worsening of oral phase may be related to the motor and cognitive deterioration of patients at follow up documented by worsening of several clinical ratings scales. In line with this hypothesis, a significant greater proportion of patients presented clinically significant difficulties with cutlery and utensils at follow up (PSP-rs item 4). Dysfunction in different phases of swallowing appears early in different neurodegenerative diseases and a shorter latency from the onset of the PSP symptoms to development of dysphagia is associated with worse overall prognosis [4, 10].

As part of the standard of care at our center and in line with recent recommendations, all PSP patients are referred early during the disease course to a Speech Language Pathologist to detect swallowing issues and provide suggestions for food management and consistency. Notwithstanding, our patients presented a significant variation in eating habits at mid-term follow up [11].

Carrying out nutritional assessments and indicating appropriate dietary adjustments in relation to disease status are important aspects of the treatment of patients with PSP, also with the aim of supporting the caregiver in food choices [11].

Improving our knowledge on the specific dietary interventions for PSP in relation with changes in swallowing should be investigated in future studies. A targeted dietary regime, with specific protein and vitamin supplementation should be considered early during the course of the disease [12].

Recent studies focused on the role of specific dietary regimes or nutritional supplements in preventing or slowing down neurodegenerative diseases [12]. Vitamins B6, B12, vitamin D, vitamin E and folic acid were demonstrated to be a valid nutritional supplementation for AD [12]. Similarly, probiotics, particularly *Lactobacillus* spp., can improve synaptic plasticity, stimulate hippocampal neurogenesis, regulate hypothalamic- pituitary-adrenal axis and reduce the level of oxidative stress [12]. Well-designed trials are needed to understand the usefulness of supplements for PSP patients before such recommendation can be incorporated in clinical guidelines [12].

We failed to show important differences when dividing the PSP cohort based on disease phenotypes. Although we recognize that our cohort was mainly represented by PSP-RS (66.66%), once again the phenotypes of the disease did not make any difference in the proposed evaluations [5].

The main limitations of our study were the small sample size and the short follow-up. Notwithstanding, this is the first longitudinal study evaluating energy expenditure, body composition and dietary habits in PSP.

## Conclusions

At mid-term follow up, PSP patients presented reduced caloric, protein and fibers intake, regardless the presence of clinically relevant dysphagia for solids, while the amount of fats and carbohydrate remained stable. The PSP-rs is likely not adequate in assessing dysphagia in its complexity and specific clinical scales or instrumental examinations are needed. With the goal of maintaining adequate nutritional status, the administration of protein and vitamin supplements should be considered even in the absence of worsening of dysphagia documented by the corresponding PSP-rs items.

### Supplementary Information

Below is the link to the electronic supplementary material.

Supplementary file1 (DOCX 30.6 KB)

## Data Availability

The datasets generated during and/or analysed during the current study are available from the corresponding author on request.

## Abbreviations

REE: Rest energy expenditure
ALS: Amyotrophic lateral sclerosis
PSP: Progressive supranuclear palsy
FFM: Fat-free mass
BMI: Body mass index
TDEE: Total daily energy expenditure
PAL: Physical activity intensity levels
FM: Fat mass
LEDD: Levodopa equivalent daily dose
PSP-rs: PSP-rating scale
MDS-UPDRS-III: Movement Disorder Society version of the United Parkinson's disease III
S&E: Schwab and England
MOCA: Montreal Cognitive Assessment
PSP-RS: PSP Richardson’s syndrome
vPSP: Other variant syndromes of PSP
