# Supplementary material for: Longitudinal change of energy expenditure, body composition and dietary habits in Progressive Supranuclear Palsy patients
Source: Neurol Sci. 2024 Apr 19;45(9):4335–40. doi: 10.1007/s10072-024-07533-5 (PMC11306440; doi:10.1007/s10072-024-07533-5)
Supplement: Supplementary file 1 — Supplementary file1 (DOCX 30.6 KB) [file 10072_2024_7533_MOESM1_ESM.docx]

**Supplemental methods**

Body weight was calculated with a calibrated flat scale (SECA GMBH 7997021099; Germany) according to standard procedure, and height was measured with a mobile stadiometer. Body mass index (BMI) is defined as body mass divided by the square of body height and was expressed in units of kg/m^2^. Subjects were thus divided into normal weight (BMI 18.5-24.9), overweight (BMI 24.9-29.9) and obese (BMI>29.9). Waist circumference was determined with a tape measure placed at the midpoint between the last rib and the iliac crest.

Resting energy expenditure (mREE), or the number of calories required by the body to maintain vital physiological functions, was calculated with the FitMate® portable indirect calorimeter (Cosmed, Rome, Italy), which uses measurements of resting oxygen consumption (VO2). The examination is conducted by having the patient breathe for 15 minutes inside a multipurpose silicone mask with mouthpiece and filter.

The calculation of total daily energy expenditure (TDEE) is derived from the sum of mREE and metabolism during physical activity, making an estimate of the last 7 days. The mREE was measured with the indirect calorimeter as explained above. Metabolism during physical activity is given by the sum of kilocalories (kcal) consumed in the different types of activities, also considering their duration according to specific equation [1-3].

The calculation of kcal during activity is derived from a specific equation: kilocalories=MET X weight in kilograms X duration in hours.

The following were considered for the calculation of METs/hour: for sedentary occupations or sitting at rest 1.5 METs; for standing 2.5 METs; for moderate or intense manual activities 4.0 or 6.0 METs respectively; for household chores 3.3 METs; and for moderate intensity activities such as walking or intense activities such as running 3.5 and 6 METs respectively.

Moreover, the hours spent performing the various activities and the different types of tasks performed in the day were calculated with the free online Grana Padano software (www.educazionenutrizionale.granapadano.it) [3-5].

The estimated physical activity (PAL) intensity levels (sedentary or poor active or active or very active) was calculated from the ratio of TDEE to REE [6].

**Supplemental references**

1. Festi D, Colecchia A, Pini S, Scaioli E, Maffeis C, Coccheri S, Petroni ML (2009) Development and application of a simple and powerful tool for nutrition and lifestyle education for the Italian general population by general practitioners and family pediatricians. Mediterr J Nutr Metab 2:139-144.
2. Bollati V, Favero C, Albetti B, Tarantini L, Moroni A, Byun H, Motta V, Conti DM, Tirelli AS, Vigna L, Bertazzi PA, Pesatori AC (2014) Nutrients intake is associated with DNA methylation of candidate inflammatory genes in a population of obese subjects. Nutrients 6(10):4625-4639.
3. Istituto Nazionale di Ricerca per gli Alimenti e la Nutrizione (I.N.R.A.N.) (2000) Tabelle di composizione degli Alimenti. Aggiornamento 2000. EDRA Medical Publishing and New Media.
4. Festi D, Colecchia A, Pini S, Scaioli E, Maffeis C, Coccheri S, Petroni ML (2009) Development and application of a simple and powerful tool for nutrition and lifestyle education for the Italian general population by general practitioners and family pediatricians. Mediterr J Nutr Metab 2:139-144.
5. Bollati V, Favero C, Albetti B, Tarantini L, Moroni A, Byun H, Motta V, Conti DM, Tirelli AS, Vigna L, Bertazzi PA, Pesatori AC (2014) Nutrients intake is associated with DNA methylation of candidate inflammatory genes in a population of obese subjects. Nutrients 6(10):4625-4639.
6. LARN: Livelli di Assunzione di Riferimento di Nutrienti ed energia per la popolazione italiana. IV Revisione, 2014.

**Table S1. Study assessments in PSP-RS and vPSP at follow-up**

|  | **PSP-RS**  **(N=10)** | **vPSP**  **(N=5)** | **p** |
| --- | --- | --- | --- |
| *Demographic, clinical and anthropometric data* | | | |
| Age, years | 69.1 (4.43) | 71.2 (5.02) | 0.422 |
| Sex, men, n (%) | 5 (50) | 5 (100) | 0.053 |
| Disease duration, years | 3.6 (1.64) | 4.6 (2.96) | 0.409 |
| LEDD, mg/day median (IQR) | 300 (413) | 200 (350) | 0.804 |
| PSP-rs | 47.3 (8.89) | 39.8 (13.53) | 0.217 |
| MDS-UPDRS-III | 39.80 (15.74) | 46 (17.56) | 0.500 |
| S&E (IQR) | 50 (13) | 60 (50) | 0.531 |
| MOCA | 17.23 (5.64) | 17.17 (5.42) | 0.985 |
| Weight, Kg | 79.45 (12.61) | 80 (4.63) | 0.927 |
| BMI, Kg/m^2^ median (IQR) | 27.55 (7.11) | 27.64 (5.04) | 0.624 |
| Waist circumference, cm | 100.4 (8.69) | 103.4 (3.2) | 0.352 |
| *Energy expenditure* | | | |
| Measured rest energy expenditure (mREE), Kcal/day median (IQR) | 1693.0 (540) | 1752.0 (550) | 0.951 |
| Total daily energy expenditure (TDEE), Kcal/day | 2502.33 (659.04) | 2422.43 (454.52) | 0.813 |
| Physical activity level (PAL) | 1.48 (0.11) | 1.48 (0.10) | 0.974 |
| PAL intensity levels, n (%)  1.40-1.69 low  1.70-1.99 moderate  2.00-2.40 high | 7 (70)  1 (10)  0 (0) | 4 (80)  0 (0)  0 (0) |  |
| *Body composition* | | | |
| Fat free mass, Kg | 56.28 (9.78) | 64.38 (10.92) | 0.180 |
| Fat free mass index, Kg/m | 32.24 (3.97) | 37.6 (6.99) | 0.268 |
| Fat mass, Kg | 22.05 (10.73) | 15.62 (8.45) | 0.272 |
| Fat mass index, Kg/m | 13.65 (7.04) | 28.62 (6.24) | 0.219 |
| Skeletal muscle mass, Kg | 27.35 (7.43) | 33.48 (9.10) | 0.196 |
| Skeletal muscle mass index, Kg/m2 | 10.09 (1.94) | 11.42 (3.40) | 0.363 |
| Pathologic skeletal muscle mass index, n (%) | 0 (0) | 2 (40) | 0.110 |
| Body cellular mass, Kg | 28.62 (6.24) | 35.24 (15.07) | 0.263 |
| Body cellular mass index, Kg/m | 17.36 (2.65) | 20.62 (9.08) | 0.324 |
| Total body water, L | 42.36 (7.47) | 49.16 (9.17) | 0.158 |
| Total body water index, L/m | 25.75 (3.12) | 28.66 (5.77) | 0.238 |
| Extracellular water, L | 20.53 (3.39) | 22.24 (3.15) | 0.374 |
| *Dietary intake* | | | |
| Calorie intake, Kcal/day | 1695.8 (625.53) | 1694.2 (573.05) | 0.996 |
| Protein intake, g/day | 69.79 (23.89) | 66.68 (17.14) | 0.801 |
| Carbohydrates intake, %  - Sugars, % | 45.3 (6.03)  21.8 (8.71) | 50.6 (7.98)  24.6 (4.39) | 0.172  0.516 |
| Lipid intake, % median (IQR)   - SFA, % median (IQR) - PUFA, % | 36.0 (6)  11.5 (4)  5 (1.24) | 25.0 (15)  11 (2)  5.2 (3.34) | 0.124  0.144  0.866 |
| Water intake, mL/day | 1260 (455.09) | 1180 (584.80) | 0.774 |
| Fibers intake, g/day | 22.54 (9.6) | 30.12 (9.53) | 0.172 |
| Calcium intake, mg/day | 1042.0 (447.22) | 899.0 (140.83) | 0.252 |
| Iron intake, intake, mg/day | 9.76 (3.68) | 11.72 (2.6) | 0.155 |
| Zinc intake, mg/day | 9.71 (3.45) | 10.08 (1.5) | 0.412 |
| Vitamin A intake, μg/day | 1000.90 (309.991) | 1219.6 (309.991) | 0.124 |
| Vitamin D intake, μg/day | 2.44 (1.13) | 2.24 (1.22) | 0.379 |
| Vitamin E intake, mg/day | 10.81 (2.89) | 11.84 (5.50) | 0.318 |
| Vitamin B12 intake, mg/day | 4.62 (1.83) | 4.98 (1.93) | 0.365 |
| Vitamin C intake, mg/day | 179.6.88 (100.65) | 266.8 (97.59) | 0.067 |
| Folate intake, μg/day | 329.2 (119.24) | 405.4 (120.552) | 0.133 |
| Alcohol intake, g/day median (IQR) | 1.3 (11.2) | 3 (8) | 0.499 |

Data are expressed in mean (standard deviation), unless otherwise specified.

Abbreviations: PSP: Progressive Supranuclear Palsy; PSP-RS: Progressive Supranuclear Palsy with Richardson’s syndrome; PUFA: Poly-unsaturated fatty acids; SFA: Saturated fatty acids; vPSP: the other variant syndromes of Progressive Supranuclear Palsy

**Table S2. Comparison in dietary intake between PSP with and without clinically significant dysphagia for solids**

|  | **PSP without dysphagia for solid**  **(N=10)** | **PSP with dysphagia for solid**  **(N=5)** | **p** |
| --- | --- | --- | --- |
| *Demographic, clinical and anthropometric data* | | | |
| Age, years | 70.7 (5.07) | 68 (3) | 0.297 |
| Sex, men, n (%) | 7 (70) | 3 (60) | 0.699 |
| Disease duration, years median (IQR) | 3 (3) | 5 (3) | 0.129 |
| LEDD, mg/day | 280 (234.75) | 450 (254.95) | 0.221 |
| PSP-rs | 41.80 (11.09) | 50.80 (7.98) | 0.132 |
| MDS-UPDRS-III | 42.70 (17.54) | 40.20 (14.16) | 0.787 |
| S&E median (IQR) | 50 (30) | 50 (25) | 0.347 |
| MOCA | 15.70 (5.54) | 20.21 (3.88) | 0.130 |
| Weight, Kg | 81.2 (11.38) | 76.5 (8.38) | 0.431 |
| BMI, Kg/m^2^ median (IQR) | 27.5 (4.52) | 27.6 (9) | 1 |
| Waist circumference, cm | 103.5 (6.38) | 97.2 (7.95) | 0.169 |
| *Energy expenditure* | | | |
| Measured rest energy expenditure (mREE), Kcal/day | 1794 (360.62) | 1461 (417.99) | 0.133 |
| Total daily energy expenditure (TDEE), Kcal/day | 1799.4 (361.39) | 1464.2 (418.21) | 0.131 |
| Physical activity level (PAL) median (IQR) | 1.47 (0.17) | 1.44 (0.19) | 0.797 |
| PAL intensity levels, n (%)  < 1.40 sedentary  1.40-1.69 low active  1.70-1.99 active  2.00-2.40 very active | 2 (20)  8 (80)  0  0 | 1 (20)  3 (60)  1 (20)  0 | 1  0.409  0.143  1 |
| *Body composition* | | | |
| Fat free mass, Kg | 60.75 (11.40) | 56.34 (9.35) | 0.476 |
| Fat free mass index, Kg/m | 36.08 (5.99) | 34.28 (3.89) | 0.558 |
| Fat mass, Kg | 19.46 (11.83) | 20.28 (7.36) | 0.892 |
| Fat mass index, Kg/m median (IQR) | 11.5 (5.5) | 13 (8.65) | 0.797 |
| Skeletal muscle mass, Kg | 30.16 (8.72) | 28.41 (8.28) | 0.721 |
| Skeletal muscle mass index, Kg/m2 | 10.66 (2.93) | 10.39 (1.81) | 0.856 |
| Pathologic skeletal muscle mass index, n (%) | 2 (20) | 0 (0) | 0.255 |
| Body cellular mass, Kg | 37.71 (12.19) | 27.88 (4.94) | 0.419 |
| Body cellular mass index, Kg/m | 19.40 (6.91) | 16.96 (2.19) | 0.464 |
| Total body water, L | 45.85 (9.09) | 42.88 (7.78) | 0.550 |
| Total body water index, L/m | 27.20 (4.89) | 26.06 (3.25) | 0.652 |
| Extracellular water, L | 21.05 (3.20) | 21.30 (3.83) | 0.900 |
| *Dietary intake* | | | |
| Calorie intake, Kcal/day | 1725.30 (464.63) | 1635.2 (847.99) | 0.883 |
| Protein intake, g/day | 69.71 (18.49) | 66.84 (28.47) | 0.816 |
| Carbohydrates intake, % median (IQR)  - Sugars, % | 43 (8)  20.90 (7.46) | 47 (14)  26.40 (6.80) | 0.804  0.190 |
| Lipid intake, %   - SFA, % - PUFA, % median (IQR) | 33.80 (6.56)  10.90 (2.18)  5 (4) | 35.80 (6.83)  12.60 (3.05)  4 (2) | 0.592  0.233  0.254 |
| Water intake, mL/day median (IQR) | 1400 (900) | 1400 (500) | 0.768 |
| Fibers intake, g/day | 27.48 (9.21) | 20.24 (10.57) | 0.194 |
| Calcium intake, mg/day | 994.80 (375.04) | 993.4 (412.82) | 0.995 |
| Iron intake, intake, mg/day | 11.22 (3.03) | 8.8 (3.87) | 0.205 |
| Zinc intake, mg/day median (IQR) | 11.80 (3.4) | 11.2 (6.6) | 0.513 |
| Vitamin A intake, μg/day | 1074.2 (276.30) | 1073 (471.98) | 0.995 |
| Vitamin D intake, μg/day | 2.14 (1.06) | 2.84 (1.20) | 0.271 |
| Vitamin E intake, mg/day median (IQR) | 13.2 (3.4) | 10.5 (3.1) | 0.310 |
| Vitamin B12 intake, mg/day | 5.01 (2.06) | 4.2 (1.13) | 0.434 |
| Vitamin C intake, mg/day median (IQR) | 219 (113) | 136 (127) | 0.679 |
| Folate intake, μg/day | 368. 80 (116.75) | 326.20 (138.49) | 0.541 |
| Alcohol intake, g/day | 6.17 (5.85) | 1.6 (2.32) | 0.053 |

Data are expressed in mean (standard deviation), unless otherwise specified.

Abbreviations: PSP: Progressive Supranuclear Palsy; PSP-RS: Progressive Supranuclear Palsy with Richardson’s syndrome; PUFA: Poly-unsaturated fatty acids; SFA: Saturated fatty acids; vPSP: the other variant syndromes of Progressive Supranuclear Palsy
